# Altered amygdala activation during face processing in Iraqi and Afghanistani war veterans

**DOI:** 10.1186/2045-5380-1-6

**Published:** 2011-10-12

**Authors:** Alan N Simmons, Scott C Matthews, Irina A Strigo, Dewleen G Baker, Heather K Donovan, Arame Motezadi, Murray B Stein, Martin P Paulus

**Affiliations:** 1 Veterans Affairs San Diego Healthcare System, 3350 La Jolla Village Drive, San Diego, CA 92161, USA; 2 University of California San Diego, 9500 Gilman Drive, La Jolla, CA 92093, USA; 3 Center of Excellence in Stress and Mental Health, VASDHS, 3350 La Jolla Village Drive, San Diego, CA 92161, USA; 4 Research Service & VA Mental Illness Research, Education and Clinical Center, VASDHS, 3350 La Jolla Village Drive, San Diego, CA 92161, USA

## Abstract

**Background:**

Exposure to combat can have a significant impact across a wide array of domains, and may manifest as post-traumatic stress disorder (PTSD), a debilitating mental illness that is associated with neural and affective sequelae. This study tested the hypothesis that combat-exposed individuals with and without PTSD, relative to healthy control subjects with no history of PTSD or combat exposure, would show amygdala hyperactivity during performance of a well-validated face processing task. We further hypothesized that differences in the prefrontal cortex would best differentiate the combat-exposed groups with and without PTSD.

**Methods:**

Twelve men with PTSD related to combat in Operations Enduring Freedom and/or Iraqi Freedom, 12 male combat-exposed control patients with a history of Operations Enduring Freedom and/or Iraqi Freedom combat exposure but no history of PTSD, and 12 healthy control male patients with no history of combat exposure or PTSD completed a face-matching task during functional magnetic resonance imaging.

**Results:**

The PTSD group showed greater amygdala activation to fearful versus happy faces than both the combat-exposed control and healthy control groups. Both the PTSD and the combat-exposed control groups showed greater amygdala activation to all faces versus shapes relative to the healthy control group. However, the combat-exposed control group relative to the PTSD group showed greater prefrontal/parietal connectivity with the amygdala, while the PTSD group showed greater connectivity with the subgenual cingulate. The strength of connectivity in the PTSD group was inversely related to avoidance scores.

**Conclusions:**

These observations are consistent with the hypothesis that PTSD is associated with a deficiency in top-down modulation of amygdala activation by the prefrontal cortex and shows specific sensitivity to fearful faces.

## Background

Soldiers exposed to combat in Operations Iraqi Freedom (OIF) and Enduring Freedom (OEF) are at high risk for post-traumatic stress disorder (PTSD) [1]. PTSD is an aversive reaction to a life-threatening, emotionally salient event [2] that is associated with increased mortality and morbidity [3]. The majority of those who experience such an event have a substantial stress response [4] that is characterized by activation in physiological and neuroendocrine systems [5-8]. Such stress responses are associated with hyperactivation in the insula and amygdala [9,10] brain structures that are involved in processing emotional information. Amygdala activation has been strongly linked to negative affective states in fear processing [11-13] and PTSD [14-17]. A number of studies have successfully used face tasks to probe affective circuits such as the amygdala to better understand affective symptomology in PTSD [18-23]. However, amygdala activation has not been as consistent in PTSD as in other anxiety groups [10]. While a number of studies have shown amygdala hyperactivation in individuals with combat-related PTSD versus healthy controls (HC) with no history of PTSD or combat exposure [20,23-25], a similar number of studies have shown amygdala hyperactivation in individuals with combat-related PTSD versus individuals with combat exposure but no PTSD [21,26-28]. Other studies have shown amygdala hyperactivation in individuals with combat exposure but not PTSD relative to individuals with no history of combat exposure or PTSD [29]. Although these findings suggest strongly that PTSD is related to amygdala hyperactivation, it can also be suggested that the experience of emotional trauma in and of itself may relate to significant differences in the functioning of emotional processing circuits.

Exposure to combat where there is a risk of death (in other words, Criterion A for the diagnosis of PTSD; [30]) can have significant psychiatric or cognitive repercussions [31] even when it does not result in PTSD. However, one important difference between those exposed to trauma who develop PTSD versus those who do not may be in the increased avoidance of aversive experiences and emotions [32]. This maladaptive response to aversive emotions following trauma may enhance and maintain symptoms of PTSD [33] by diminishing the likelihood of fear extinction [34].

Recent neural models of PTSD and trauma exposure suggest that the functional networks associated with the amygdala may be of similar importance to understanding emotional processing as the amygdala itself [35]. These theories posit that PTSD is, in part, a manifestation of ineffective top-down modulation of the amygdala and limbic circuitry by the prefrontal cortex [15,35]. This model has been proposed as a mechanism for the depersonalization seen in PTSD [36]. For example, it has been shown that reduced functional connections between the amygdala and prefrontal cortex relate to increased levels of depersonalization following emotional trauma, suggesting that impaired functioning of this prefrontal modulatory network may be related to clinical symptoms in traumatized individuals [37].

The use of multiple control groups can be effective in separating the contributions of combat exposure and PTSD. Specifically, a multiple control group design is useful for testing the hypothesis that trauma disrupts emotional circuits relevant to face processing, and that the subsequent development of PTSD is related to less engagement of frontal top-down circuitry [15,35].

Two recent papers investigated the effects of trauma and PTSD during functional magnetic resonance imaging (fMRI) through comparison of PTSD, trauma-exposed and HC subjects. Using a cognitive inhibition task, Falconer and colleagues found greater frontal activation in the trauma-exposed and HC individuals versus the PTSD subjects, and greater parahippocampal activation in PTSD versus HC (but not trauma control) individuals [38]. New and colleagues compared how these three groups relate when downregulating emotion during performance of a negative cognitive reappraisal task and found that both HC and trauma controls relative to PTSD subjects showed greater activation of frontal circuitry. However, they did not find significant differences in amygdala activation between the groups [39]. The authors of this paper posit that the 'trauma-exposed groups may engage a more distributed cortical network in the control of emotion' (p. 662) than HCs, suggesting that trauma-exposed controls show greater limbic and frontal activation in the control of emotion. Both of these studies used cognitive tasks in groups with non-combat trauma exposure. In addition, positron emission tomography (PET) studies have used the three-group model to dissociate the biomarkers of PTSD and trauma. Phan and colleagues suggest that both trauma and PTSD groups show activations in the amygdala but that the PTSD group differentially modulates the ventral medial frontal gyrus [40]. Britton and colleagues showed that the dorsal medial frontal gyrus was less active in patients with PTSD while was there was greater ventromedial prefrontal activation [41]. These findings suggest the importance of frontal circuitry in trauma response and resilience. However, the degree to which these findings translate to a sample with combat trauma during performance of a task that probes affective brain circuits is unknown. Delineation of the effects of combat exposure and PTSD will help increase understanding of possible mechanisms of resilience or vulnerability to PTSD after exposure to trauma.

In prior studies, using a simple face-matching task, we and others have identified clinically meaningful differences in amygdala activation in groups with mood and anxiety disorders [42-44], and shown significant changes in response to psychopharmacological intervention [45,46]. This simple face-matching task has also been successful in delineating differences in functionally connected networks in psychiatric populations [43]. While face tasks do not use trauma-related stimuli and do not directly provoke re-experiencing symptoms in PTSD, they do require appraisal of social emotions, thus they appear to provide a method to measure affective circuitry in a theoretically and clinically meaningful way.

In the current study, we collected fMRI data in combat-exposed veterans with and without PTSD, as well as in healthy participants, during performance of a face-matching task that reliably activates the amygdala [43,44] in an effort to understand how neural response in affective circuitry could help delineate the effects of trauma from PTSD. Based on the literature described above, we hypothesized that PTSD individuals would show greater amygdala activation during a face matching task relative to HC participants with no history of PTSD or combat exposure. Furthermore, functional connectivity between the amygdala and frontoparietal structures, including the dorsal lateral and medial prefrontal cortex, involved in emotion modulation would be reduced in the PTSD group in contrast to the trauma-exposed groups.

## Results

### Demographic, psychiatric and behavioral results

After correcting for multiple comparisons, the groups did not differ on several demographic variables (Table 1). The PTSD and combat-exposed control (CEC) groups did not differ on combat exposure, childhood trauma severity or depression. However, the PTSD had significantly higher scores on the Clinician-Administered PTSD Scale (CAPS) total and on several subscales of the CAPS relative to the CEC group. A significant difference of condition was seen in the reaction time, but not accuracy, from the face-matching task. This difference was largely powered by the shorter reaction time to the shapes versus the faces, and mirrored results from prior analysis. No group, or group by condition, differences were seen for reaction time or accuracy (see Table 1; Supplementary Tables 2-4 in Additional File 1).

**Table 1:** Demographic, psychiatric and behavioral variables.

	PTSD		CEC		HC			
**Variable**	**Mean**	**SD**	**Mean**	**SD**	**Mean**	**SD**	**F-statistic**	***p***

**Age**	32.3	6.9	28.7	4.5	27.3	6.2	2.193	0.128
**Education**	13.9	1.7	13.8	2.2	13.5	2.0	0.065	0.938
**Ethnicity (n)**								
**Caucasian**	6		10		5		χ^2 ^= 6.20	0.185
**Hispanic**	3		2		5			
**Black**	3		0		2			
**CTQ**	39.8	5.4	42.6	6.5	-	-	0.858	0.371
**CES**	22.7	7.2	19.1	9.9	-	-	0.623	0.444
**BDI**	14.6	9.5	5.5	5.9	-	-	5.083	0.042
**CAPS-B**^**a**^	19.0	7.3	3.9	6.9	-	-	20.194	< 0.001
**CAPS-C**	25.8	5.3	4.8	8.0	-	-	40.163	< 0.001
**CAPS-D**	25.0	3.9	11.0	8.4	-	-	18.584	< 0.001
**CAPS Total**	69.8	13.2	19.7	22.2	-	-	31.497	< 0.001
**Task reaction time (ms)**							70.974^b^	< 0.001^b^
**Shape**	1029	317	965	278	919	188	1.146^c^	0.384^c^
**Angry**	1458	435	1470	455	1347	251		
**Fear**	1652	369	1625	532	1395	336		
**Happy**	1257	289	1185	336	1009	161		

^a ^CAPS was measured in eight out of twelve of the PTSD group and ten out of twelve of the CEC group; ^b^task effect; ^c^group effect. BDI = Beck Depression Inventory; CAPS = Clinician-Administered PTSD Scale; CEC = combat exposed control; CES = Combat Exposure Scale; CTQ = Childhood Trauma Questionnaire; HC = healthy control; PTSD = post traumatic stress disorder.

### Neuroimaging results (region of interest analyses)

**Figure 1:**
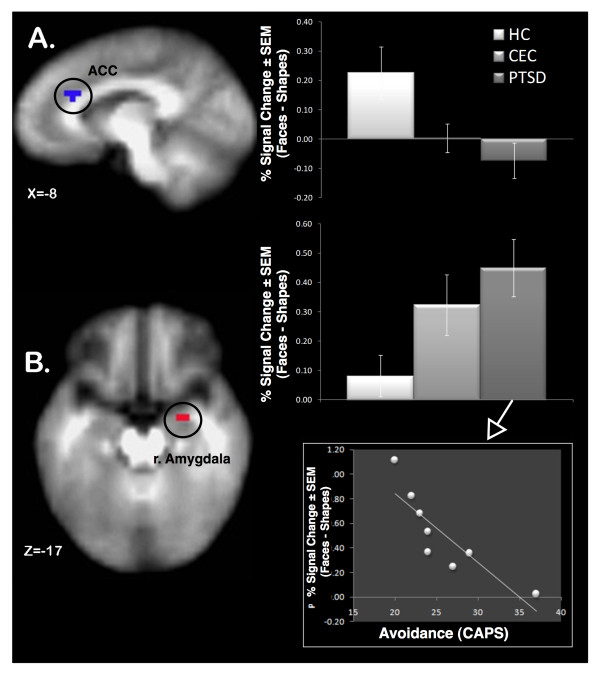
**Faces versus shape activation differences for the combat groups versus healthy controls**. **(A) **Anterior cingulate cortex and **(B) **amygdala. **(C) **Contrasts for angry-shape, fear-shape, and happy-shape for the amygdala region. **(D) **Correlation in the PTSD group between avoidance on the CAPS and face-shape. Details for the associated clusters provided in Table 2.

Clusters of significant activation were found for all three contrasts of interest. These were the effect of PTSD, measured as the task-related activity in PTSD versus CEC individuals; the effect of combat exposure, or task-related activity in PTSD and CEC individuals versus HCs; and the effect of task, which was the activity for face-matching minus shape-matching trials in all groups (PTSD, CEC and HC; see Table 2). In the PTSD-CEC contrast, the right insula was significantly more active in the PTSD group and the anterior cingulate was significantly more active in the CEC group. In the PTSD+CEC-HC contrast, we found significantly greater activation for the PTSD +CEC group in the right amygdala, whereas the left anterior cingulate was significantly more active in the HC group (see Figure 1). In the task effect, we found significant activation in the right and left amygdala, the right and left insula and right hippocampus, as well as significant deactivations in numerous clusters throughout the anterior cingulate, and insula (see Table 2). Additional post-hoc analysis was done contrasting the three groups on the fearful-happy contrast. The PTSD group had significantly greater activation in the amygdala than both groups (Table 3) while the CEC and HC groups did not differ significantly.

**Table 2:** Region of interest analysis for all faces versus shapes.

Volume	x	y	z	Region	t-statisic	df	*p*
**PTSD-CEC**							
**320**	40	-23	3	Right insula	4.03	22	< 0.001
**320**	2	12	20	Right anterior cingulate	-2.48	22	< 0.05
**(PTSD+CEC)-HC**							
**320**	-9	27	23	Left anterior cingulate	-3.11	34	< 0.005
**256**	24	-5	-18	Right amygdala	2.71	34	< 0.01
**PTSD+CE+HC**							
**3712**	5	45	0	Right anterior cingulate	-5.57	35	< 0.001
**1536**	23	-6	-18	Right amygdala	6.14	35	< 0.001
**1472**	-22	-5	-18	Left amygdala	5.30	35	< 0.001
**896**	13	33	22	Right anterior cingulate	-3.30	35	< 0.005
**576**	-39	-36	20	Left insula	-3.16	35	< 0.005
**512**	-17	41	-5	Left anterior cingulate	-3.64	35	< 0.001
**512**	-3	31	-5	Left anterior cingulate	-3.05	35	< 0.005
**448**	-40	-22	6	Left posterior insula	3.89	35	< 0.001
**384**	53	-35	18	Right insula	-4.62	35	< 0.001
**320**	41	-24	3	Right posterior insula	3.25	35	< 0.005
**256**	15	39	14	Right anterior cingulate	-3.35	35	< 0.005
**192**	44	6	-8	Right anterior cingulate	-2.46	35	< 0.05
**192**	-10	31	8	Left anterior cingulate	-2.23	35	< 0.05
**192**	12	30	12	Right anterior cingulate	-2.30	35	< 0.05
**192**	51	-20	16	Right insula	-3.26	35	< 0.005

CEC, combat exposed control; HC, healthy control; PTSD; post traumatic stress disorder group.

**Table 3:** Region of interest analysis for fearful versus happy faces.

vol	x	y	z	Region	BA	t-statistic	*p*
**PTSD > HC**							
**1152**	-36	5	2	Left insula	13	3.13	0.01
**576**	36	10	14	Right insula	13	2.38	0.05
**320**	-23	-5	-15	Left parahippocampal Gyrus/amygdala		2.56	0.05
**320**	-44	10	-3	Left insula	13	2.38	0.05
**320**	-3	21	21	Left anterior cingulate	33	2.56	0.05
**192**	-21	-1	-12	Left parahippocampal Gyrus/amygdala		2.72	0.05
**PTSD > CEC**							
**7616**	40	-3	5	Right insula	13	2.93	0.01
**7424**	1	36	14	Right anterior cingulate	32	2.93	0.01
**5376**	-39	-2	1	Left insula	13	3.09	0.01
**448**	-36	-2	16	Left insula	13	3.53	0.005
**384**	-19	-3	-14	Left parahippocampal Gyrus/amygdala		3.03	0.01
**192**	18	-8	-15	Right parahippocampal Gyrus/amygdala		3.10	0.01
**HC > CEC**							
**512**	45	-22	8	Right posterior insula	13	2.84	0.01
**448**	36	7	6	Right insula	13	2.69	0.05
**384**	-35	2	16	Left insula	13	2.67	0.05
**320**	10	31	24	Right anterior cingulate	32	2.71	0.05
**320**	-7	30	24	Left anterior cingulate	32	2.32	0.05
**PTSD+CEC+HC**							
**2624**	38	16	-1	Right insula	13	2.96	0.01
**896**	-34	19	1	Left insula	13	2.77	0.05
**640**	7	49	-4	Right anterior cingulate	32	-4.42	0.001
**384**	20	-4	-17	Right parahippocampal Gyrus/amygdala		2.62	0.05
**384**	-13	41	-3	Left anterior cingulate	32	-2.52	0.05
**384**	48	5	2	Right insula	13	2.88	0.01
**320**	-6	30	22	Left anterior cingulate	32	2.82	0.01

CEC = combat exposed control; HC = healthy control; PTSD = post traumatic stress disorder group; BA = Brodmann Area.

### Brain-behavior correlations

When inspected in the PTSD group alone, a significant inverse correlation was observed between the avoidance subscale ("C") of the CAPS and right amygdala activation in the group (PTSD+CEC-HC) contrast (Spearman's rho = -0.976, *P *< 0.001; n = 8) and the task (faces-shapes) contrast (Spearman's rho = -0.796, *P *= 0.026; n = 8). No other subscales of the CAPS correlated significantly with amygdala activations. It should be noted the fearful-happy contrast did not correlate in the group or task contrast regions of interest (ROIs; Spearman's rho = -.228, *P *= 0.588, n = 8; and Spearman's rho = -0.132, *P *= 0.756, n = 8, respectively). The avoidance subscale was selected based on prior findings within our group [47].

### Functional connectivity results

In order to examine differences within functional amygdala networks between the combat-exposed groups (PTSD versus CEC), we performed a functional connectivity analysis with the bilateral amygdala as seed regions. This analysis revealed that the sole area that the PTSD group showed greater connectivity with the right amygdala, compared to the CEC group, was in the subgenual cingulate cortex. There was no area where PTSD showed greater connectivity using the left amygdala seed. However, the CEC compared to the PTSD group showed numerous areas with significantly greater functional connections with bilateral amygdala, such as the posterior cingulate, inferior frontal, middle occipital, and superior temporal gyri (Table 4; Supplementary Figures 1 and 2 in Additional File 1).

**Table 4:** Functional connectivity results from amygdala task seed regions of interest differences between PTSD and CEC.

Seed	vol	x	y	z	Region	BA	t-statistic	*p*
**Right amygdala**								
**PTSD > CEC**								
	**1728**	6	16	2	Subgenual cingulate gyrus/caudate	25	3.027	< 0.01

**CEC > PTSD**								
	**59200**	2	-46	28	posterior cingulate gyrus	31	-3.814	< 0.001
	**11200**	-49	2	4	Left superior temporal gyrus	22	-5.137	< 0.001
	**5248**	34	37	12	Right middle frontal gyrus	10	-4.162	< 0.001
	**2624**	30	-70	13	Right middle occipital gyrus	30	-3.527	< 0.005
	**2368**	51	-35	-5	Right middle temporal gyrus	20	-3.727	< 0.001
	**2240**	-36	23	-26	Left superior temporal gyrus	38	-5.271	< 0.001
	**1280**	-35	-38	-33	Left cerebellum		-3.281	< 0.005
	**1152**	-26	46	2	Left superior frontal gyrus	10	-3.699	< 0.001
	**1152**	-12	-21	4	Left thalamus		-2.290	< 0.05
	**1024**	9	-38	14	Right posterior cingulate	29	-3.107	< 0.005
	**1024**	50	-39	26	Right inferior parietal lobule	13	-3.539	< 0.005
	**832**	28	0	-37	Right uncus	20	-3.207	< 0.005

**Left amygdala**								

**PTSD > CEC**								
	**-**	-	-	-	-	-	-	-
**CEC > PTSD**								
	**4096**	-4	-76	26	Left occipital gyrus	18	-2.983	< 0.01
	**3840**	48	21	18	Right inferior frontal gyrus	45	-2.714	< 0.05
	**2496**	4	1	41	Right cingulate gyrus	24	-2.375	< 0.05
	**1664**	-49	8	12	Left precentral gyrus	44	-3.668	< 0.001
	**1600**	58	-50	2	Right middle temporal gyrus	21	-3.450	< 0.005
	**1536**	48	-47	30	Right supramarginal gyrus	40	-3.156	< 0.005
	**1408**	-28	13	-24	Left superior temporal gyrus	38	-2.558	< 0.05
	**1408**	-3	-50	31	Left precuneus	31	-2.464	< 0.05
	**1408**	13	-41	39	Right posterior cingulate gyrus	31	-3.087	< 0.005
	**1024**	32	-74	16	Right middle occipital gyrus	19	-2.601	< 0.05
	**896**	-6	-79	-15	Left occipital gyrus	18	-2.809	< 0.01
	**832**	-47	0	25	Left inferior frontal gyrus	9	-2.971	< 0.01

CEC = combat exposed control; HC = healthy control; PTSD = post traumatic stress disorder group; BA = Brodmann Area.

## Discussion

This experiment yielded three main findings. First, individuals with PTSD and CECs without PTSD showed significantly greater right amygdala activation during an affective face-matching task when compared to HCs without PTSD or combat exposure in the all faces minus shapes contrast, while only the PTSD group had significantly higher activation in the amygdala for the fearful-happy contrast when contrasted with the CEC and HC groups. Second, in the PTSD group, task-related amygdala activation showed a significant inverse correlation with the severity of avoidance symptoms (as measured by the CAPS). Third, in the CEC compared to the PTSD group, the amygdala showed greater functional connectivity with frontal and parietal regions. Taken together, these findings are consistent with the hypothesis that individuals with combat exposure show a generalized increased limbic activation (in other words, in the amygdala) versus controls without combat exposure; and that among combat-exposed individuals, greater connectivity between the amygdala and frontal cortex may be associated with greater resilience to the development of PTSD. Furthermore, these findings suggest that those with PTSD may attempt to 'turn down' amygdala activation through avoidance.

Increased emotional reactivity in the amygdala has been linked to depression [48], anxiety [49,50], PTSD [14] and genetic vulnerability to psychiatric disorders [51,52]. Using the same task that was administered in the current study, we observed similar findings in major depressive disorder [42,43], trait anxiety [44] and victims of domestic violence [19]. The amygdala has been a relatively robust measure of trauma-related reactivity, especially in studies using PET scans [41,53-56]. However, amygdala findings have been somewhat split in the PTSD literature in fMRI studies, potentially due to avoidance or similar mechanisms associated with PTSD [9,10] and/or the exact contrasts used in the analysis. In the current study, we found that amygdala activation was greater in the PTSD group in the fearful-happy contrast. This suggests that this contrast does have specific relevance to PTSD. However, this activation did not correlate to symptom severity. In contrast, we found that combat exposure was associated with amygdala hyperactivation irrespective of PTSD in the faces versus shape contrast. Despite similarly increased amygdala activation to face-shape processing in both combat-exposed groups in the current study; it may be that different functional mechanisms and neural networks are utilized in the PTSD and CEC groups to modulate amygdala hyperactivation. Specifically, the PTSD group may potentially use a psychological mechanism (such as avoidance) while preliminary evidence suggests that the CECs use a cognitive or neural regulatory mechanism (in other words, top-down modulation). These findings further extend previous work, showing weaker connectivity with amygdala functioning in PTSD versus HCs [19,57], into the comparison with trauma-exposed controls. Even though amygdala activation was similar across trauma groups for when all faces were taken together, when fearful faces were separately contrasted with happy faces the PTSD group showed a significant difference with both control groups in more dorsal regions of the amygdala. These findings replicate prior data in PTSD literature [19-21,24,27] and suggest that PTSD individuals show a specific sensitivity to fearful faces that is not seen in trauma controls. These findings are in line with relative consistency of greater sensitivity in the amygdala with regard to aversive versus positive stimuli (such as fearful versus happy faces). The capacity to modulate the amygdala can therefore be an effective way to control affective responses to aversive stimuli.

There is strong evidence of a reciprocal relationship between activation in the medial prefrontal cortex and amygdala in PTSD in combat veterans [54]. This work also showed that regional blood flow in the amygdala correlated positively with PTSD symptom severity while blood flow in the medial prefrontal cortex correlated negatively with PTSD symptom severity. A similar reciprocal relationship between the subgenual cingulate and amygdala has been shown in depression [58], as well as in normal controls where the rostral cingulate and lateral prefrontal cortex in conjunction appear to regulate the amygdala during processing of faces [59,60]. Furthermore, animal and human data appear to converge on a model in which successful fear extinction depends on the functionality of this network [61]. Taken together, these studies suggest that this amygdala-prefrontal cortex network may play an important role in trauma exposure such that those who experience trauma and have more robust connections between the amygdala and the prefrontal cortex are less likely to develop PTSD [11,15,35].

It is important to note that, although the CEC group showed greater functional connections between the amygdala and numerous regions across the brain, only the subgenual cingulate cluster was found to be more functionally connected to the amygdala in the PTSD group. In a prior study using the same task, we observed similar patterns of functional connectivity, whereby the dorsal cingulate was less functionally connected with the amygdala and the subgenual cingulate was more functionally connected with the amygdala in depressed versus non-depressed individuals [43]. Decreased connectivity between the amygdala and the dorsal anterior cingulate cortex (ACC) has been observed in individuals with current depression relative to controls [62,63], and connectivity increased significantly in depressed individuals following treatment [62]. These findings are in line with evidence that altered functional activity of the amygdala and cingulate may represent a biomarker for psychiatric stress. In a separate study that investigated face processing, contrasting PTSD due to domestic violence and HCs, we found that the subgenual cingulate, in contrast to the insula, showed greater connectivity in the PTSD group [57]. We interpret these findings as an indication of a recursive connection that further fosters, rather than regulates, regional activation. In line with this hypothesis, prior research has indicated that the subgenual/rostral cingulate is an important region for modulation of amygdala reaction [60,64,65]. The subgenual cingulate has specifically been outlined as being a primary area in the response to sad faces as well as being a biomarker for negative mood [49,66-68]. Several PET and fMRI studies indicate that neural substrates such as the amygdala and subgenual cingulate, which are critical for emotion processing, are hyperactive in individuals with major depressive disorder both at rest [58] and during emotional tasks [69-72]. Conversely, brain structures such as the dorsal ACC and middle/superior frontal gyrus, which are involved in the cognitive control of behavior [73] and emotion [74], are hypoactive in individuals with depression both at rest [58] and during cognitive tasks [75]. Anatomical studies in animal have identified efferent projections from the ACC to the amygdala [76]. In connectivity studies in humans, the subgenual ACC showed strong connections with the amygdala and medial temporal lobe [77]. It has been suggested that subgenual ACC activation is observed when individuals attend to their internal emotional states [78]. Related evidence suggests that this structure is deactivated by performing difficult cognitive tasks that require an external focus of attention and prompt inhibitory control processes [79,80]. This further suggests that the CECs are enacting a more cognitive approach to the situation than the PTSD group.

Modulatory control of behavioral-affective responses such as avoidance has been posited as an important mechanism for regulating emotional responses in individuals with PTSD in both psychological [81] and neural [82] models. Avoidance symptoms inversely correlate with brain activation in task relevant emotional processing areas [83] as well as in individuals with PTSD compared to non-traumatized [47] and traumatized controls [84]. In addition, individuals with PTSD experiencing greater dissociative symptomology, as opposed to greater re-experiencing symptoms, showed attenuation of connectivity to a wide area of brain regions important in affective processing [85]. Diffusion tensor imaging studies in PTSD suggest that the anatomical integrity of connecting fibers in the medial and posterior corpus may be compromised in children with PTSD with a history of childhood maltreatment [86]. Similar reductions in posterior white matter integrity were found in the right superior longitudinal fasciculus OEF/OIF combat veterans who develop psychiatric disorders, such as major depressive disorder, after blast-related concussion [42]. Taken together, these findings support the notion that avoidance symptoms in PTSD may be associated with reduced amygdala connectivity with frontal structures. This provides confirmatory evidence for the hypothesis that individuals with PTSD may show less neural/cognitive control and resort to increased symptom avoidance.

Other important regions of differential activation were observed in the current study. Specifically, the anterior cingulate gyrus was more active in the HCs versus the CECs as well as in CECs versus the individuals with PTSD. Also, the insula was more active in the PTSD patients versus the CECs. Several prior studies have reported anterior cingulate gyrus hypoactivity in PTSD [87-89], which has been hypothesized to relate to the degree to which those with PTSD engage in emotional tasks. This hypothesis is congruent with the current findings. Increased insula activation has also been associated with PTSD [9,87]. This may suggest that individuals with PTSD have an impaired ability to maintain homeostasis via integration of physiological and emotional information [90]. Our prior work indicates that those with PTSD may have greater ability to alter insula activation in the face of a changing affective environment [91].

This study has several notable limitations. First, while a complete structured clinical interview was completed in all individuals, six of the 24 participants did not complete the CAPS. Therefore, it was not possible to determine if the correlation with avoidance symptoms remained significant across the complete sample. Second, the task used was not, nor was it intended to be, a provocation task. Thus trauma response is not being modeled in the current design; rather, everyday emotion processing patterns are being assessed. In addition, the face versus shape contrast focuses on face-processing in addition to a fearful versus happy contrast. The fearful versus happy faces contrast was done primarily to verify that the current dataset is behaving in accordance with prior literature. The faces versus shape contrast was selected due to its robust findings as well as the importance of face-processing in daily functioning. While these two conditions are very different, the group differences in areas of affective processing areas are the primary focus of this paper. This contrast is being used to probe the social affective processing of judging facial expressions without focus on valence. Third, the results of this study require replication given the modest size of the sample. Fourth, since the initiation of this study much has been learned about the importance of comorbid head injury. Unfortunately, this was not measured or controlled for in the current study.

## Conclusions

PTSD, like all mental disorders, has a multifactorial etiology. The current study highlights the potential impact of trauma exposure on neural response to affectively relevant stimuli, regardless of whether or not this trauma experience results in PTSD. Prior studies investigating the neuropsychological sequelae of OEF/OIF combat suggest that trauma exposure may explain more of the variance in cognitive performance than PTSD alone [92]. Following the experience of trauma, the development and maintenance of PTSD may relate to the way in which individuals attempt to regulate this hyperactivation. Here, we find that those without PTSD activate more top-down modulatory areas, while those who acquire PTSD show less neural connectivity with emotion regulation centers but instead exhibit avoidance. It is unclear from the current findings if these effects represent vulnerabilities, or rather are results of the trauma experience.

## Methods

### Subjects

Twelve men with combat-related PTSD, 12 men with combat-exposure without PTSD (CEC), and 12 HC men with no history of combat exposure or PTSD completed a face-matching task during fMRI. The groups were demographically matched (Table 1). Subjects were excluded if they had a lifetime history of alcohol or substance dependence, a history of alcohol or substance abuse within 30 days of study participation, irremovable ferromagnetic material, claustrophobia, bipolar disorder or schizophrenia. PTSD subjects with other comorbid anxiety or mood disorders, such as major depressive disorder, were included as long as PTSD was the clinically predominant disorder. The CEC group had experienced a PTSD 'Criterion A' event but did not have current or past PTSD. The CEC group was free of Axis I psychiatric diagnosis. The HC group had no military experience, no history of a PTSD 'Criterion A' event, and no history of current or past Axis I psychiatric diagnoses. PTSD and CEC participants were recruited from the VA San Diego Healthcare System. All participants gave informed written consent to participate in this study, which was approved by the University of California San Diego Human Research Protection Program and the Research and Development Committee at the VA San Diego Healthcare System.

### Psychiatric measures

All subjects completed a Structured Clinical Interview for Diagnostic and Statistical Manual of Mental Disorders edition 4 (SCID) [93] including the PTSD module, the Beck Depression Inventory (BDI) [94], Childhood Trauma Questionnaire [95] and the Combat Exposure Scale [96]. In addition, eight PTSD and ten CEC participants performed an additional session during which the CAPS [97] was completed. The presence of PTSD was determined by the results of the PTSD module of the SCID, and the diagnosis was confirmed by a board certified psychiatrist (DGB/MPP) and confirmed by the CAPS when available.

### Stimulus and apparatus

During fMRI, participants performed a face-matching task, with a comparison condition of shape matching (described in more detail in [43,44]). Each five-second trial consisted of a target face (on the top of the computer screen) and two probe faces (on the bottom left and bottom right of the computer screen). Participants were instructed to match the emotion (angry, fearful or happy) of the target and probe faces. The trials were block designed, such that each block consisted of six consecutive trials in which the target face was angry, fearful or happy. During the shape-matching control condition, subjects were presented with an analogous configuration of ovals and circles, and were instructed to match the shape of the target and probe stimuli. Each block of the faces and shapes was presented three times in pseudorandom order, and a fixation cross was interspersed between each block. Reaction time (RT) data were obtained for each trial. Due to device errors, RT was not recorded from three participants (one from the PTSD and two from the CEC group); however this did not affect subject feedback or the subject experience.

### Image acquisition

fMRI data were collected during the task using a Signa EXCITE 3.0 Tesla GE (General Electric, New York, NY, USA) scanner (T2-weighted echo planar imaging, repetition time (TR) = 2000 ms, echo time (TE) = 32 ms, field of view (FOV) = 230 × 230 mm^3^, 64 × 64 matrix, yielding 30 2.6 mm axial slices with a 1.4 mm gap, 256 scans). For anatomical reference, a high resolution T1-weighted image (spoiled grass sequence; inversion time = 450 ms, TR = 8 ms, TE = 4 ms, FOV = 250 × 250 mm^3^, flip angle = 12°, 172 sagittally acquired slices, approximately 1 mm^3 ^voxels) was obtained during the same session.

### fMRI analysis pathway/image processing

#### Single subject

All structural and functional image processing was done with the Analysis of Functional Neuroimages software package (AFNI, Bethesda, MD, USA). The echoplanar images were realigned to a base using a Fourier transform using the AFNI program 3dvolreg and then time-corrected for slice acquisition order. Preprocessed time series data for each individual were analyzed using a multiple regression model. For this model, the four orthogonal regressors of interest were (1) happy, (2) angry, (3) fearful and (4) circle/oval sensorimotor condition. These regressors were convolved with a modified gamma variate function to account for the delay and the dispersion of the hemodynamic response of the BOLD-fMRI signal. Additionally, five nuisance regressors were used to account for residual motion (roll, pitch and yaw) and to eliminate slow signal drifts (baseline and linear trend). These nine regressors were applied to the AFNI program 3dDeconvolve in order to calculate the estimated voxelwise response amplitude. The linear contrast of all faces versus shapes was used for maximal power in subsequent analyses. In addition, the fearful-happy faces were analyzed to provide better comparison of these results with prior literature. To account for individual variation of anatomical landmarks, a Gaussian filter with 4 mm full width at half maximum was applied to the voxelwise percent signal change data and then resampled to a 4 mm^3 ^voxel dimension.

#### Group analysis

Data for each subject were normalized to Talairach coordinates. A voxel-based two-way ANOVA was performed with group (PTSD, CEC and HC) as a fixed factor and participants as a random factor within AFNI on the faces-shapes contrast. An additional, post-hoc ANOVA was performed on the fearful-happy contrast. *A priori *ROIs, including the amygdala, hippocampus, cingulate cortex and insula, were used as masks, as these regions are of interest in PTSD [98], stress [99] and face processing [100]. Based on these four areas, a voxel-wise *a priori *probability of 0.05 was determined via simulations using the AFNI function AlphaSim [101], which resulted in a corrected cluster-wise activation probability of 0.05 using a minimum volume of 192 mm^3 ^and three connected voxels (for an amygdala or hippocampus cluster), or 320 mm^3 ^and five connected voxels (for a cingulate or insular cortex cluster). Small volume corrections were used to inspect areas that may not have been of sufficient size to meet minimum whole brain cluster size corrections. Using the thresholds and cluster sizes defined above, the corrected voxel-wise probabilities are as follows: amygdala *P *< 0.002, hippocampus *P *< 0.0002, cingulate cortex *P *< 0.0008 and insular cortex *P *< 0.0008. The areas of interest were superimposed on each individual's voxel-wise percent signal change brain image. Stereotactic coordinates of the ROIs were based on standardized atlas locations [102]. Only the activations within the regions of interest that survived the volume and voxel correction criteria were extracted and used for further analysis.

### Functional connectivity analysis

To examine between-group differences in functional connectivity within the amygdala, we performed a modified Psycho-Physiological Interaction analysis that has been used previously [103]. Prior to this analysis, the individual raw signal datasets were: (a) band-pass filtered (0.009 Hz < ƒ < 0.08 Hz), (b) corrected for slice-dependent time shifts, (c) corrected for interleaved acquisition, (d) corrected for rigid body head motion, and (e) warped to conform to the Talairach atlas. Individual time courses in the processed raw signal datasets were extracted from seed ROIs in left and right amygdala. These two seeds were identified based on the task-related activation in all 36 subjects related to all face-matching minus all shape-matching trials (see Table 2). This region was selected to provide a task relevant region that was minimally biased between groups. A total of twelve regressors were then entered into a multiple regression against the voxel-based time series: the regressors of interest being the interaction between seed region time-course and faces blocks, along with 11 nuance regressors: three movement (roll, pitch, yaw) regressors, the four task regressors described previously, the entire time course of the seed region, the entire time course of the white matter, and baseline and linear trend regressors. The deconvolution analyses for left and right amygdala were run separately. The resulting correlation coefficients were then calculated for each voxel, providing correlation maps of the relationships between the time courses of activation in the voxels within the seed ROI's and all voxels. The Fisher's Z transforms of these correlation maps were then warped to conform to the Talairach atlas and a Gaussian blur of 4 mm full width half maximum was applied to allow for a between-groups comparison. Data for each subject were normalized to Talairach coordinates. A voxel-based two-way ANOVA was performed with group (PTSD, CEC and HC) as a fixed factor and participants as a random factor within AFNI on the Fisher Z for each amygdala. The following planned linear contrasts were performed: (1) PTSD-CEC, to determine the effects of PTSD, (2) PTSD+CEC-HC, to determine the effects of trauma exposure and (3) PTSD+CEC+HC, to determine the task effects. A voxel-wise *a priori *probability of 0.05 was determined via simulations using the AFNI function AlphaSim, as above, which resulted in a corrected cluster-wise activation probability of 0.05 using a minimum volume of 768 mm^3 ^in a whole brain analysis.

### Statistical analysis

All behavioral and secondary analyses were carried out with StatistiXL 1.8 (StatistiXL, Nedlands, Western Australia). Continuous demographic and performance variables such as age, education, RT and accuracy were compared between the groups in a fixed-factor ANOVA. Ethnicity was compared using a chi-squared test across all three groups. Clinical measures, such as BDI and CAPS scores, were compared between PTSD and CEC groups using a two-sample t-test.

## List of abbreviations

ACC: anterior cingulate cortex; AFNI: analysis of functional neuroimages; ANOVA: analysis of variance; BDI: Beck Depression Inventory; CAPS: Clinician-Administered PTSD Scale; CEC: combat exposed control; fMRI: functional magnetic resonance imaging; FOV: field of view; HC: healthy control; OEF: Operation Enduring Freedom; OIF: Operation Iraqi Freedom; PET: positron emission tomography; PTSD: post-traumatic stress disorder; ROI: region of interest; RT: reaction time; SCID: Structured Clinical Interview for DSM-IV; TE: echo time; TR: repetition time.

## Competing interests

The authors declare that they have no competing interests.

## Authors' contributions

ANS was involved in the conception and design of the study as well as acquisition, analysis and interpretation of data. SCM was involved in interpretation of the data. IAS was involved in analysis and interpretation of the data. DBG was involved in the acquisition of the study data. HKD and AM were involved in the acquisition of the data for the study. MBS and MPP participated in the conception and design of the study. All authors read, contributed to and approved the final manuscript.

## Supplementary Material

Additional file 1 **Supplementary Data**. Additional information to aid in the interpretation of the results (as listed below). Supplementary Table 1. Whole brain group contrasts for the faces versus shape. Supplementary Table 2. Task accuracy data group by condition. Supplementary Table 3. Within subjects analysis of task performance data. Supplementary Table 4. Between subject analysis of task performance data. Supplementary Figure 1. Functional connectivity results from left amygdala task seed region of interest differences between PTSD and CEC. Supplementary Figure 2. Functional connectivity results from right amygdala task seed region of interest differences between PTSD and CEC. Click here for file
