# Supplementary material for: Altered amygdala activation during face processing in Iraqi and Afghanistani war veterans
Source: Biol Mood Anxiety Disord. 2011 Oct 12;1:6. doi: 10.1186/2045-5380-1-6 (PMC3384263; doi:10.1186/2045-5380-1-6)

**Supplementary Data**

**Supplementary Table 1.** Whole brain group contrasts for the faces versus shape.

| **Contrast** | **Volume** | **x** | **y** | **z** | **Within** | **BA** | **T-value** |
| --- | --- | --- | --- | --- | --- | --- | --- |
| **PTSD+CEC>HC** |  |  |  |  |  |  |  |
|  | - | - | - | - | - | - | - |
| **PTSD+CEC<HC** |  |  |  |  |  |  |  |
|  | 1728 | -34 | 53 | -1 | Left Middle Frontal Gyrus | 10 | -2.78 |
|  | 1152 | -19 | 61 | 6 | Left Superior Frontal Gyrus | 10 | -2.86 |
|  | 1088 | -49 | 20 | 17 | Left Inferior Frontal Gyrus | 45 | -2.72 |
|  | 768 | -7 | 21 | 51 | Left Superior Frontal Gyrus | 6 | -2.61 |
| **PTSD>HC** |  |  |  |  |  |  |  |
|  | - | - | - | - | - | - | - |
| **PTSD<HC** |  |  |  |  |  |  |  |
|  | 1088 | -36 | 52 | -2 | Left Middle Frontal Gyrus | 10 | -2.73 |
| **PTSD>CEC** |  |  |  |  |  |  |  |
|  | 2368 | 40 | 15 | -18 | Right Inferior Frontal Gyrus | 38 | 2.71 |
|  | 2240 | 57 | -53 | 3 | Right Middle Temporal Gyrus | 21 | 2.89 |
|  | 1600 | 45 | -25 | -7 | Right Middle Temporal Gyrus | 22 | 2.84 |
|  | 1408 | 45 | 10 | 21 | Right Inferior Frontal Gyrus | 9 | 2.65 |
|  | 1344 | -1 | -53 | -25 | Left Cerebellum |  | 3.07 |
|  | 1152 | 44 | -16 | 26 | Right Postcentral Gyrus | 6 | 2.82 |
|  | 1088 | 9 | -36 | -33 | Right Cerebellum |  | 2.95 |
|  | 896 | -30 | -51 | -26 | Left Cerebellum |  | 2.60 |
|  | 832 | 27 | -78 | 36 | Right Precuneus | 19 | 2.76 |
| **PTSD<CEC** |  |  |  |  |  |  |  |
|  | 768 | -36 | -62 | -5 | Left Fusiform Gyrus | 37 | -3.05 |
| **CEC<HC** |  |  |  |  |  |  |  |
|  | 1344 | -49 | 20 | 16 | Left Inferior Frontal Gyrus | 45 | -2.62 |
|  | 832 | 35 | 7 | 40 | Right Middle Frontal Gyrus | 9 | -2.69 |
| **CEC>HC** |  |  |  |  |  |  |  |
|  | - | - | - | - | - | - | - |

Note. The whole brain is clustered to 768mm^3^. T-values are averaged across voxels within regions.

**Supplementary Table 2.** Task accuracy data group by condition.

| **Condition** |  | **PTSD** | |  | | **CEC** | |  | | **HC** | |  |
| --- | --- | --- | --- | --- | --- | --- | --- | --- | --- | --- | --- | --- |
|  | Mean | | SD | | Mean | | SD | | Mean | | SD | |
| **Angry** | 100.0 | | 0.0 | | 100.0 | | 0.0 | | 96.8 | | 9.6 | |
| **Fear** | 99.1 | | 3.0 | | 97.5 | | 4.6 | | 96.8 | | 7.7 | |
| **Happy** | 95.9 | | 8.0 | | 96.3 | | 5.2 | | 95.8 | | 7.5 | |
| **Shape** | 96.5 | | 7.1 | | 95.6 | | 9.0 | | 96.9 | | 3.5 | |

**Supplementary Table 3.** Within subjects analysis of task performance data.

| **Effect** | **Wilks Lambda** | **Ho Df** | **Error Df** | **F** | **Prob.** |
| --- | --- | --- | --- | --- | --- |
| **Reaction Time** |  |  |  |  |  |
| **Repeat** | 0.116 | 3 | 28 | 70.974 | >0.001 |
| **Repeat*Group** | 0.793 | 6 | 56 | 1.146 | 0.348 |
| **Accuracy** |  |  |  |  |  |
| **Repeat** | 0.869 | 3 | 26 | 1.305 | 0.294 |
| **Repeat*Group** | 0.919 | 6 | 52 | 0.376 | 0.891 |

**Supplementary Table 4**. Between subject analysis of task performance data.

| **Source** | **Type III SS** | **Df** | **Mean Sq.** | **F** | **Prob.** |
| --- | --- | --- | --- | --- | --- |
| **Reaction Time** |  |  |  |  |  |
| **Group** | 846867 | 2 | 423434 | 1.147 | 0.331 |
| **Error** | 11070414 | 30 | 369014 |  |  |
| **Accuracy** |  |  |  |  |  |
| **Group** | 0.004 | 2 | 0.002 | 0.358 | 0.702 |
| **Error** | 0.161 | 28 | 0.006 |  |  |

Supplementary Figure 1. Functional connectivity results from Left Amygdala Task seed region of interest differences between PTSD and CEC.


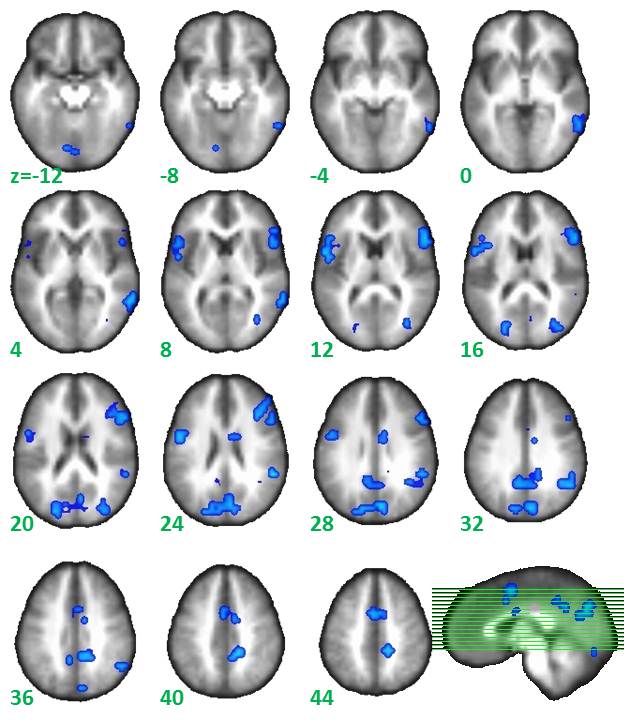


Supplementary Figure 2. Functional connectivity results from Right Amygdala Task seed region of interest differences between PTSD and CEC.


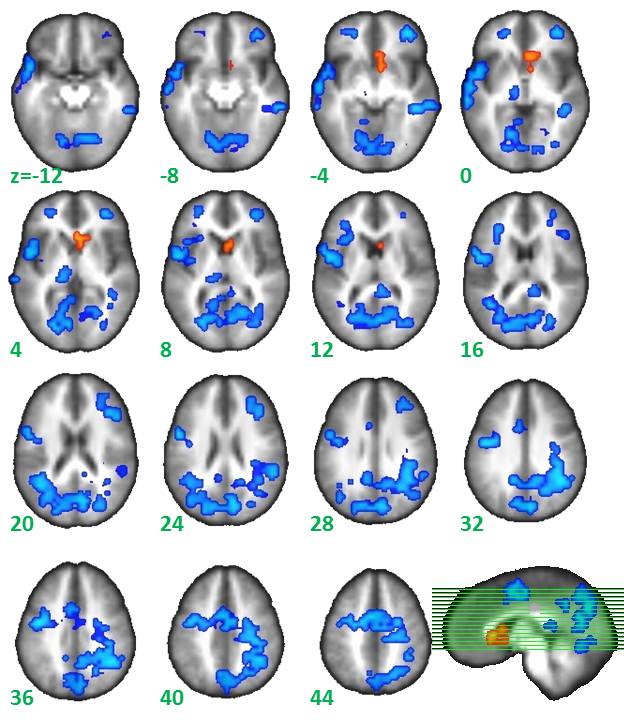

Supplement: Additional file 1 — Supplementary Data. Additional information to aid in the interpretation of the results (as listed below). Supplementary Table 1. Whole brain group contrasts for the faces versus shape. Supplementary Table 2. Task accuracy data group by condition. Supplementary Table 3. Within subjects analysis of task performance data. Supplementary Table 4. Between subject analysis of task performance data. Supplementary Figure 1. Functional connectivity results from left amygdala task seed region of interest differences between PTSD and CEC. Supplementary Figure 2. Functional connectivity results from right amygdala task seed region of interest differences between PTSD and CEC. [file 2045-5380-1-6-S1.DOC]
